# Expression, Purification, and Characterisation of South African Cassava Mosaic Virus Cell-to-Cell Movement Protein

**DOI:** 10.3390/cimb44060186

**Published:** 2022-06-15

**Authors:** Nikita Nankoo, Ikechukwu Anthony Achilonu, Marie Emma Christine Rey

**Affiliations:** Plant Biotechnology Research Laboratory, School of Molecular and Cell Biology, University of the Witwatersrand, Johannesburg 2050, South Africa; 389088@students.wits.ac.za (N.N.); ikechukwu.achilonu@wits.ac.za (I.A.A.)

**Keywords:** South African cassava mosaic virus (SACMV), cell-to-cell movement protein, ANS, mant-ATP, extrinsic ANS fluorescence, intrinsic tryptophan fluorescence

## Abstract

South African cassava mosaic virus (SACMV) is a circular ssDNA bipartite begomovirus, whose genome comprises DNA-A (encodes six genes) and DNA-B (encodes BC1 cell-to-cell movement and BV1 nuclear shuttle proteins) components. A few secondary and tertiary structural and physicochemical characteristics of partial but not full-length begomovirus proteins have been elucidated to date. The full-length codon-optimised SACMV BC1 gene was cloned into a pET-28a (+) expression vector and transformed into expression host cells *E. coli* BL21 (DE3). The optimal expression of the full-length BC1-encoded movement protein (MP) was obtained via induction with 0.25 mM IPTG at an OD_600_ of ~0.45 at 37 °C for four hours. Denatured protein fractions (dialysed in 4 M urea), passed through an IMAC column, successfully bound to the nickel resin, and eluted using 250 mM imidazole. The protein was refolded using stepwise dialysis. The molecular weight of MP was confirmed to be 35 kDa using SDS–PAGE. The secondary structure of SACMV MP presented as predominantly β-strands. An ANS (1-anilino-8-naphthalene sulphonate)-binding assay confirmed that MP possesses hydrophobic pockets with the ability to bind ligands such as ANS (8-anilino-1-naphthalenesulphonic acid). A 2′ (3′)-N-methylanthraniloyl-ATP (mant-ATP) assay showed binding of mant-ATP to MP and indicated that, while hydrophobic pockets are present, MP also exhibits hydrophilic regions. Intrinsic tryptophan fluorescence indicated a significant conformational change in the denatured form of BC1 in the presence of ATP. In addition, a phosphatase assay showed that MP possessed ATPase activity.

## 1. Introduction

South African cassava mosaic virus (SACMV) belongs to the family *Geminiviridae* and genus *Begomovirus*, whose members infect a wide range of plant hosts [1,2]. SACMV is composed of a bipartite genome (single-stranded circular DNA-A and DNA-B components) [1]. DNA-A ORFs of bipartite begomoviruses encode three proteins in the antisense direction—namely, the Rep-associated (AC1), replication enhancer Ren (AC2), and transcriptional activator TrAP (AC3) proteins [3]. Additionally, an overlapping AC4 ORF encodes for a putative host RNA-silencing suppressor protein. Conversely, in the sense direction, the structural coat protein (CP) is encoded by AV1 [3], and AV2 is a reported RNA-silencing suppressor and pathogenicity determinant [4]. DNA-B BC1 ORF encodes the cell-to-cell movement protein (MP) from the complementary strand, whereas the nuclear shuttle protein (NSP) is encoded from the virion sense strand by the BV1 ORF [3]. In plants, viruses have to travel through the cell wall via the plasmodesmata [5], and the MP is required for cell-to-cell and long-distance movement through its interaction with the NSP [6]. Geminiviruses also utilise host membranes such as the endoplasmic reticulum (ER) and cytoskeleton to traffic from the nucleus to the plasmodesmata [7]. There are two models suggested for the active role of geminivirus MP and NSP during cell-to-cell transport. The ‘couple-skating’ model suggests that the MP binds to the ssDNA/dsDNA–NSP complex on the cytoplasmic side of nuclear membranes or microsomal vesicles and subsequently traffics the nucleoprotein complex into the proximal cell along the endoplasmic reticulum, and via microtubules (forms the desmotubule) and filamentous actin through the plasmodesmata [8,9]. The ‘relay-race’ model suggests that, after NSP-mediated nuclear export, viral ssDNA or dsDNA associates with the MP, which further transports the viral DNA-MP through the plasmodesmata into an adjacent cell, with the assistance of other host accessory proteins [8,9].

The deduced 307 amino acid sequence of the SACMV MP [1] and functional studies [9,10] have informed a putative model of the MP protein structural domains—namely, the N-terminus and central anchor domains that putatively are both required for localisation to the cell periphery, and a C-terminal oligomerisation domain. The nuclear shuttle protein (NSP)-binding site is predicted to locate in the central region of the MP [10]. The secondary and tertiary structures of geminiviral MPs have not yet been elucidated. Since the MP of SACMV is a novel protein in that little homology exists with other characterised proteins in the database (NCBI), the elucidation of the protein structure is crucial to understanding the function of the protein in the cytoplasmic movement of the geminivirus DNA complex, and transit from cell to cell. Since the MP does not have any predicted enzymatic function, but rather the binding capability to other cellular proteins, this study was not focused on kinetics but rather on the elucidation of the basic structural characteristics related to protein–protein interactions. The molecular weight, as well as secondary and tertiary structures (protein folding) of the full-length SACMV MP, was characterised for the first time in this study.

## 2. Materials and Methods

### 2.1. Vector Construction

The MP gene sequence (923 nucleotides/307 amino acids [Figure S1]) was sent to GenScript Inc, Piscataway, NJ, USA, where the sequence was codon harmonised for expression into *E. coli* and cloned into expression vector system pET-28a (+) (Figure S2) using *Nde*I and *Not*I restriction sites. The codon harmonisation was carried out via a single nucleotide change (cysteine to guanine) in the codon translating for Alanine (Figure S4).

### 2.2. Recombinant Protein Expression

*E. coli* BL21 (DE3) cells were transformed with the codon-harmonised MP sequence in a pET-28a (+) vector. Single colonies were cultured overnight into SOB media comprising 5% (*w*/*v*) yeast extract (Sigma-Aldrich, St. Louis, MO, USA), 20% (*w*/*v*) tryptone (Sigma-Aldrich, St. Louis, MO, USA), 0.5% (*w*/*v*) NaCl (Sigma-Alrich, USA), and 1 M KCl (Merck, Darmstadt, Germany), supplemented with 1 M MgCl_2_ (Sigma-Aldrich, St. Louis, MO, USA), 1 M MgSO_4_ (Sigma-Aldrich-USA, St. Louis, MO, USA), and 50 μg mL^−1^ kanamycin (ThermoFisher Scientific, New York, NY, USA) at 37 °C, shaking at 200 rpm. The overnight cultures were diluted 1/10 with fresh SOB media supplemented with 1 M MgCl_2_, 1 M MgSO_4_, and 50 μg mL^−1^ kanamycin at 37 °C, shaking at 200 rpm until the OD_600_ was approximately 0.45. The cultures were then induced with IPTG to a final concentration of 0.25 mM and cultured for an additional 4 h (37 °C, 200 rpm). Cells were harvested via centrifugation (20,000× *g*, 10 min, 4 °C). Approximately 1 g of wet cell pellet was obtained per one hundred millilitres, and the pellet was resuspended in lysis buffer (50 mM Tris, 5% glycerol (*v*/*v*), and 50 mM NaCl; pH 7.5), followed by 6 cycles of sonication (30 s on and 30 s off). Following centrifugation at 20,000× *g* for 25 min at 4 °C, the supernatant (soluble fraction) and pellet (insoluble fraction) were separated.

### 2.3. Protein Denaturation, Purification and Refolding, and Purity Assessment

The insoluble cell fraction was subject to denaturation with 8 M urea in 50 mM Tris- HCl (pH 8.5) and 0.05% NaN_3_ (pH 7.5). The cell solution was subjected to overnight solubilisation via dialysis in 4 M urea (1 L of 4 M urea, 20 mM sodium phosphate, 0.5 M NaCl, and 10 mM imidazole; pH 7.5). The solubilised cell fraction was further subjected to purification using a gravity flow column comprising a nickel-charged NTA Profinity™ IMAC resin (Bio-Rad, Hercules, CA, USA). The IMAC column was equilibrated with dialysate (4 M urea solution). To remove any unbound protein, the resin was washed with 4 M urea, 20 mM sodium phosphate, 0.5 M NaCl, and 25 mM imidazole (pH 7.5), and eluted with 4 M urea containing 250 mM imidazole (pH 7.5). Fractions containing eluted proteins were pooled and subject to refolding via stepwise dialysis (three changes: first change, 16 h; 50 mM Tris-HCl, pH 10; 0.5 M l-arginine, 2% (*v*/*v*); Triton X-100 (Merck, Darmstadt, Germany); and 10 mM DTT; remaining changes, 16 h each; 50 mM Tris-HCl, pH 10; and 0.5 M l-arginine). The protein concentration following protein refolding was determined spectrophotometrically using a theoretically estimated molar extinction coefficient of 31,650 M^−1^ cm^−1^.

All expressed protein fractions and all eluted protein fractions were prepared with 2X SDS buffer (added in a 1:1 ratio) and run on a 12% SDS–PAGE mini-PROTEAN gels (TGX Stain-Free™ Fastcast™ acrylamide kit Bio-Rad Laboratories, Inc. (Hercules, CA, USA)). Based on the SDS–PAGE results, column fractions containing the purified protein of high purity (no contamination) (eluted fractions 1–4) were pooled together and refolded via stepwise dialysis, as described above.

### 2.4. Secondary Structure Content Determination

Far-UV circular dichroism (Far-UV CD) measurements of MP were performed at 20 °C in a Jasco J1500 spectrometer. Briefly, the protein sample was diluted to a final concentration of 5 µM using ultra-pure deionised water, which resulted in a final Tris buffer containing 0.5 mM Tris-HCl (pH 7.5). Three independent spectra were collected for both the protein sample (5 µM MP in 0.5 mM Tris-HCl, pH 7.5) and the buffer (as blank). All spectra were corrected for using the Tris-HCl buffer as a baseline. The raw data were converted to mean residue ellipticity [*Θ*] (mdeg cm^2^ dmol^−1^) using the following formula:

$$\left[\Theta \right]=\frac{100\theta }{cnl}$$

where *c* is the concentration in g/L, *n* is the number of residues, and *l* is the path length in cm.

The estimation of secondary structure of BC1 was estimated using the CONTINL algorithm in the Dichroweb online server [11].

### 2.5. Extrinsic Fluorescence ANS Binding Assay

The extrinsic ANS fluorescence spectra for the SACMV MP were measured with a Jasco FP-6300 spectrofluorometer. A 20 mM stock of ANS was resuspended in 100 mM sodium phosphate (pH 6.5), 1 mM EDTA, and 0.02% NaN_3_, and stored in the dark. The concentration of ANS was determined using UV–Vis absorbance at 350 nm. All measurements were performed in a 50 mM Tris-HCl (pH 10) and 0.5 M l-arginine buffer. Each protein sample (triplicate) was equilibrated in the buffer, and 10 µM protein was used for each measurement. In order to determine what occurs in denatured proteins, measurements were also performed in 8 M urea (Sigma-Aldrich, St. Louis, MO, USA) in 50 mM Tris-HCl (pH 8.5) and 0.05% (*w*/*v*) NaN_3_ (pH 7–7.5). Each sample was excited at 380 nm, and three emission spectra between 400 and 600 nm were collected and averaged.

### 2.6. Intrinsic Fluorescence Mant-ATP Binding Assay

The ability of the hydrophobic sites of the MP to bind mant-ATP was determined using fluorescence spectroscopy. The fluorescence spectra were measured using a Jasco FP-6300 spectrofluorometer. A 100 µM stock of an original 5 mM stock of mant-ATP (Sigma-Aldrich, St. Louis, MO, USA) was resuspended in refolding buffer (50 mM Tris-HCl, pH 10; and 0.5 M l-arginine) and in 8 M urea (8 M Urea in 50 mM Tris-HCl, pH 8.5; and 0.05% NaN_3_, pH 7–7.5). Per 100 µM of mant-ATP, 35 µM of protein was used (ratio 20:7). Each sample was excited at 360 nm, and three emission spectra between 400 and 600 nm were captured and averaged.

### 2.7. Phosphatase Activity Assay

To determine if the MP has phosphatase activity, an assay kit (Sigma-Aldrich, St. Louis, MO, USA) was used to determine the amount of free phosphate released. Experiments were prepared and measured as per the manufacturer’s instructions. All measurements were recorded at 620 nm on a Multiskan spectrophotometric plate reader. All background absorbance was corrected by subtracting background values from the standard phosphate absorbance.

### 2.8. Intrinsic Tryptophan Fluorescence

Intrinsic tryptophan fluorescence was carried out in order to determine if the protein was refolded and if there were any conformational changes in the presence and absence of ATP. Measurements were performed in the presence and absence of ATP and for the native and denatured forms of the protein. Six samples were analysed using a Jasco FP-6300 spectrofluorometer. Tryptophan residues were excited at 280 nm, and fluorescence spectra were recorded between 300 and 500 nm. Each spectrum is the average of three accumulation replicates in 50 mM Tris-HCl (pH 7.5), containing 0.5 mM l-arginine or 8 M urea. Background fluorescence (free unbound tryptophan residues) was corrected by subtracting background values from the actual protein spectra (protein bound to tryptophan residues).

## 3. Results

### 3.1. Recombinant Protein Expression Optimisation

**Figure 1:**
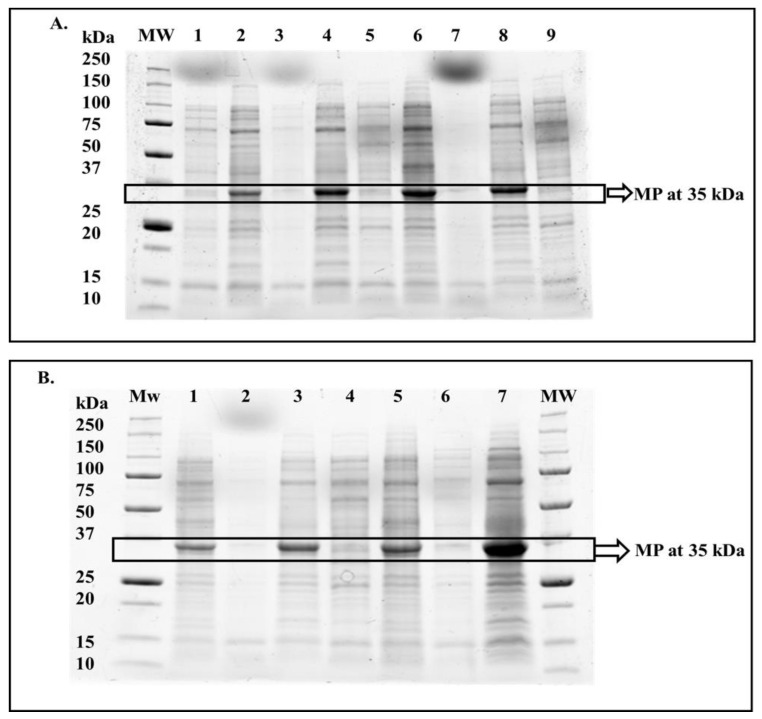
SDS–PAGE gels (12%) showing expression of SACMV MP (35 kDa including the His tag) in non-induced and induced (0.25 mM IPTG at 37 °C) fractions for 1–2 h (**A**) and 3–4 h (**B**) (**A**) Mw: molecular weight marker (Bio-Rad unstained ladder); Lanes 1: soluble, non-induced, 1 h; 2: soluble, induced, 1 h; 3: insoluble, non-induced, 1 h; 4: insoluble, induced, 1 h; 5: soluble, non-induced, 2 h; 6: soluble, induced, 2 h; 7: insoluble, non-induced, 2 h; 8: insoluble, induced, 2 h; 9: soluble, non-induced, 3 h. (**B**) Mw: molecular weight marker; Lanes 1: soluble, induced, 3 h; 2: insoluble, non-induced, 3 h; 3: insoluble, induced, 3 h; 4: soluble, non-induced, 4 h; 5: soluble, induced, 4 h; 6: insoluble, non-induced, 4 h; 7: insoluble, induced, 4 h.

The optimal expression conditions for this protein were successfully obtained in this study by varying induction temperature (4, 15, 20, 25, and 37 °C), IPTG concentration (0, 0.25, 0.50, 0.75, and 1 mM), and induction period (0–24 h). Based on the results, it was demonstrated that SACMV MP is predominantly insoluble (Figure 1A,B) and is expressed under non-induced and induced conditions. While some soluble protein can be seen (lane 7, Figure 1), this protein concentration was insufficient to continue with downstream applications post-protein purification. The optimal expression conditions for the insoluble MP were 37 °C for 4 h and induction via 0.25 mM IPTG (lane 7, Figure 1B). The estimated monomeric molecular mass of MP deduced from the SDS–PAGE gels was 35 kDa.

### 3.2. Protein Purification and Refolding

**Figure 2:**
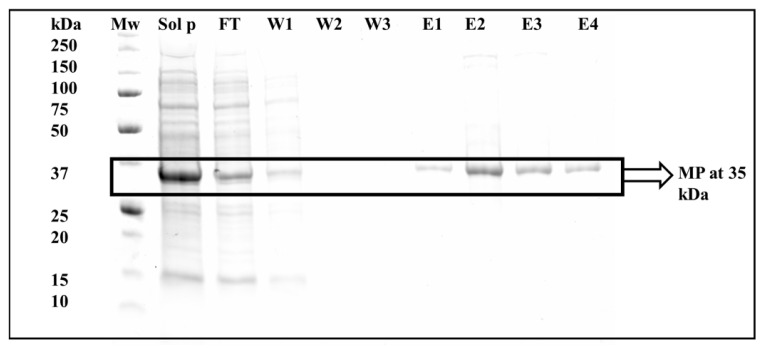
A 12% SDS–PAGE gel showing the elution fractions of MP from an IMAC column before refolding. Mw: unstained molecular weight marker protein standards. Lanes Sol p: protein fraction after solubilisation with 4 M urea; FT: flow-through fraction; W1: wash fraction 1; W2: wash fraction 2; W3: wash fraction 3; E1: protein elution fraction 1; E2: protein elution fraction 2; E3: protein elution fraction 3; E4: protein elution fraction 4.

Affinity chromatography is a technique commonly selected for the purification of proteins. A number of His-tagged proteins are often purified by means of affinity chromatography [11]. For this study, gravity-flow affinity chromatography was carried out using a nickel-charged resin. The MP was bound to the affinity column using nickel before refolding (Figure 2). Eluted MP was collected in fractions 6 and 9 (Figure 2 lanes 6–9). The introduction of 25 mM of imidazole removed unbound and loosely bound proteins off the charged column, and the final elution of the His-tagged protein occurred with the addition of 250 mM of imidazole to the nickel-charged column.

### 3.3. Secondary Structure Characterisation

**Figure 3:**
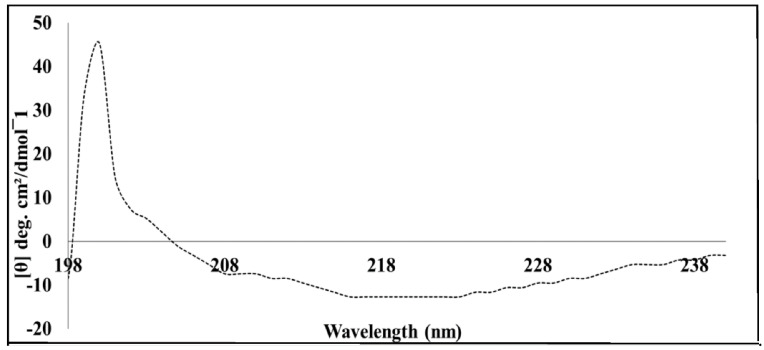
Far-UV CD spectrum of SACMV BC1. Signals were collected using 5 μM of protein in 0.5 mM Tris-HCl buffer, pH 7.5. The spectrum indicates that the protein displays a secondary structure which is predominantly β-strand.

Far-UV CD spectroscopy (between 180 and 250 nm) estimates the secondary structure content of proteins and peptides and determines the fraction of α-helices and β-strands with respect to the amino acids involved in unordered forms in an amide backbone [12]. The far-UV CD was utilised in order to determine the predominant secondary structure of the MP with the assistance of the Dichroweb server, applying the CONTINL1 algorithm. The CD spectra (Figure 3) confirmed that the MP secondary structure is predominantly (57.2%) β-strand, and data analysed and retrieved from Dichroweb confirmed this finding (Figure S3).

### 3.4. Extrinsic ANS Fluorescence Binding Assay

**Figure 4:**
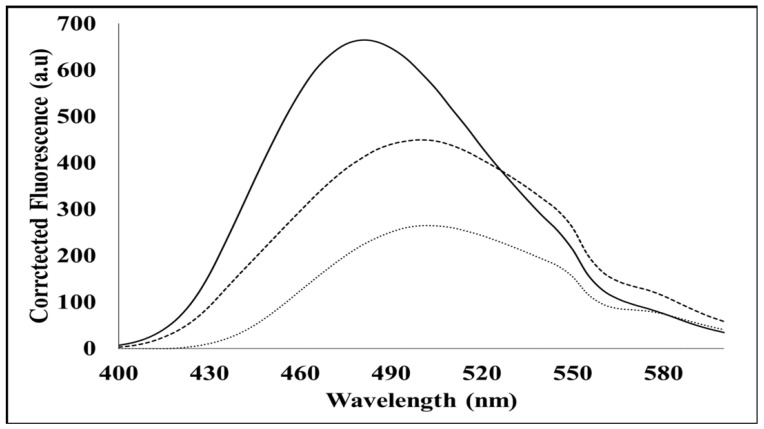
A spectrum showing extrinsic ANS fluorescence. Spectra of 200 µM ANS (star dashes) bound to 10 µm SACMV MP (solid line) and 8 M urea + 10 µM MP (dashes).

The tertiary structure of MP was examined with the aim of identifying possible hydrophobic sites in the protein. Figure 4 shows a significant shift in quantum yield and simultaneous blue shift (λ_max_) from 510 nm to 480 nm for MP (native form). This indicates that MP provides ANS a non-polar environment upon binding, suggesting that MP possesses hydrophobic binding pockets in its native form. The denatured MP showed a significant shift in quantum yield but did not show a blue shift (λ_max_) from 510 nm to 480 nm (λ_max_ remained at 510 nm) (Figure 4). This indicates that the denatured form of MP has no binding pockets available to bind ANS, compared with its native counterpart.

### 3.5. Intrinsic Fluorescence: Mant-ATP Binding Assay

**Figure 5:**
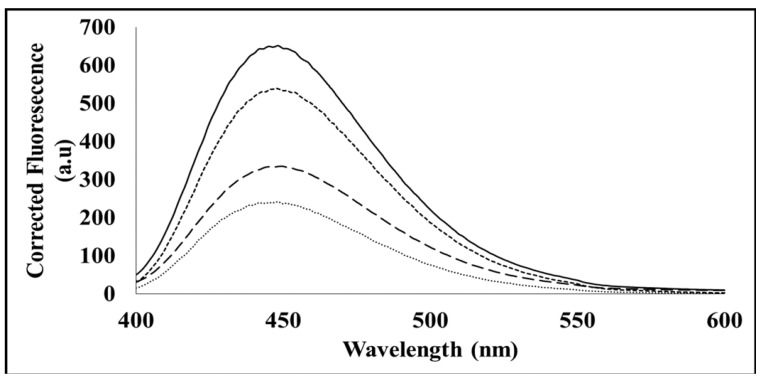
Extrinsic MANT- ATP fluorescence spectra of 100 µm MANT ATP (long dashes) bound to 10 µm MP (short dashes), 8 M urea (solid line), and 10 µm MP + 8 M urea (star dashes).

The ATP binding assay uses mant-ATP, in which the ATP molecule displays fluorescence at 360 nm and reveals the degree to which a protein binds ATP [13,14]. When excited at lower wavelengths, free mant-ATP displays low levels of fluorescence; however, when bound to a target such as the MP, a significant peak emerges [13,14]. A peak displaying a maximum of approximately 440 nm is characteristic of the quenching of intrinsic fluorescence of tryptophan and tyrosine residues and the transfer of this fluorescent energy to the mant moiety bound to ATP [13]. Fluorescence spectra results (Figure 5) demonstrate that the native form of MP, while containing hydrophobic binding sites which bind to the mant-ATP, also contains some hydrophilic regions. This conclusion was derived from the observed increase in the quantum yield, and lack of blue shift (λ_max_) in the spectra between the buffer and the native protein. In the denatured form (presence of 8 M urea), SACMV MP was unable to bind the mant-ATP.

### 3.6. Phosphatase Assay

**Figure 6:**
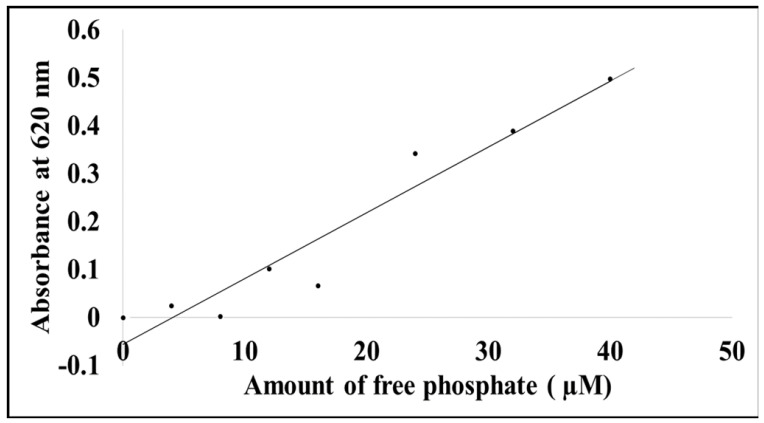
Standard curve showing the varying amount of free phosphate released at 620 nm.

The phosphatase assay was used to determine whether SACMV MP was able to hydrolyse ATP to form ADP and Pi or AMP and PPi [15]. Results showed that the MP released approximately 62 µM of free phosphate, indicating that the protein indeed possesses phosphate activity and is functional (Figure 6). The amount of inorganic free phosphate was determined from the equation of the standard curve plot of phosphate standards.

### 3.7. Intrinsic Tryptophan Fluorescence

**Figure 7:**
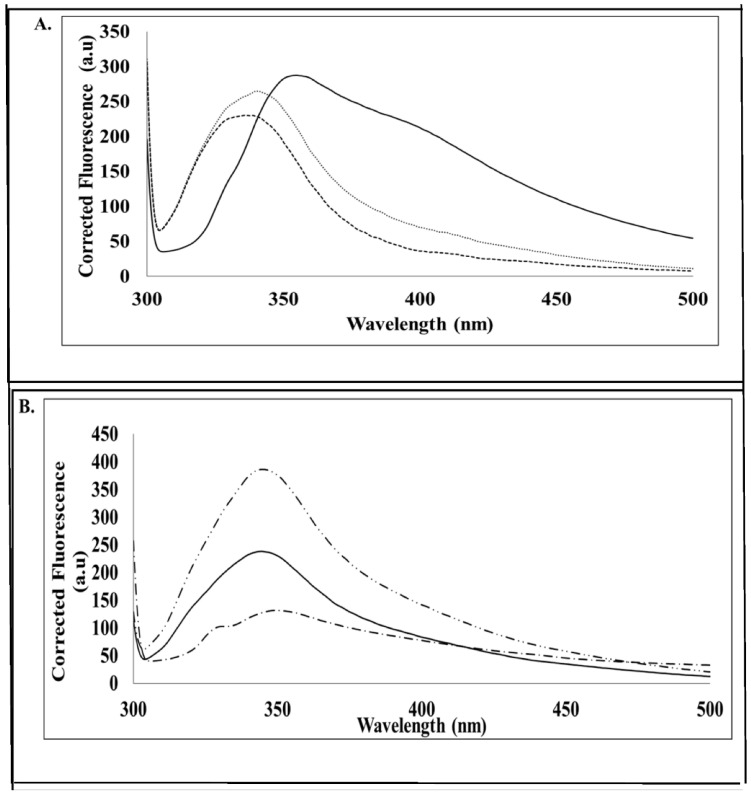
Intrinsic tryptophan fluorescence spectra: (**A**) spectra of 10 µM MP protein in the absence of ATP (star dashes) and bound to 10 µM MP in the presence of ATP (long dashes). The sample buffer (50 mM Tris-HCl, pH 7.5, containing 0.5 mM l-arginine) spectrum signal is represented by the solid line. Each spectrum is the average of three accumulations of three replicate samples; (**B**) spectra of 8 M urea (long and short dashes), 10 µM MP (denatured in 8M Urea) in the absence of ATP (solid line), and in the presence of ATP (dashes and dots).

Tryptophan fluorescence in this study was utilised for the purpose of monitoring the conformational changes in MP and is also used to monitor the successful refolding of proteins. SACMV MP contained three tryptophan residues. Figure 7A shows a blue shift (λ_max_) from 375 nm to 345 nm for native MP in the absence of ATP and native MP in the presence of ATP even though there is a significant drop in quantum yield, compared with the buffer control. Figure 7B shows an increase in quantum yield for denatured MP in the absence and presence of ATP, while no significant blue shift can be seen when compared with the urea buffer alone. The changes in the emission spectra of tryptophan occur in response to conformational transitions, substrate binding, denaturation, or subunit association [14]. These spectra results indicate that, in the presence of ATP, the native form of the MP may not undergo much conformational change. However, in the denatured form in the presence of ATP, the MP underwent a significant change in conformation. Based on the differential spectral profiles in terms of the quantum yield obtained and the slight shifts in where the quantum yield shifts were obtained between the native and denatured proteins, it can be confirmed that the protein indeed refolded.

## 4. Discussion

While low temperatures usually favour soluble protein expression [16,17,18], this was not the case for the insoluble SACMV MP. According to Burgess-Brown et al. (2008), codon optimisation increases heterologous protein expression in bacteria such as BL21 (DE3), and high expression levels of the insoluble expressed protein were obtained (Figure 1A,B). Viral proteins are notoriously difficult to purify and solubilise due to the abundance of disordered regions, leading to poorly defined secondary structure elements [19]. The Rep protein N-terminus of tomato yellow leaf curl virus (TYLCV) and the Rep protein C-terminus of tomato leaf curl Gujarat virus (ToLCGuV) have been partially characterised [20,21]. In both these studies, the soluble protein was attained only by means of expression and characterisation of a truncated (single terminus) region, as opposed to the full-length protein. This study is the first to characterise a full-length geminiviral protein. It is proposed that the lack of soluble SACMV full-length MP could be due to the bacterial *E. coli* host chosen. Bacteria such as *E. coli*, while preferred due to their fast growth rate, are also known for possible misfolding and precipitation of heterologous proteins [12,22]. An alternate expression host, yeast is a well-studied eukaryotic system used for the expression of proteins but is more difficult to work with because cell growth is slower, and the level of expression is not always high. Additionally, post-translational modifications may differ in vitro and in planta. However, working with yeast is advantageous, as the system offers a high level of intracellular expression and secretes a few of its own proteins, making expression products easier to purify for downstream characterisation [12]. While it is notable that no other attempts to express soluble geminiviral proteins in yeast have been reported in the literature, it is suggested that the BC1 gene be codon-optimised for expression in a yeast host such as *Pichia pastoris*.

Intrinsically disordered proteins or proteins with intrinsically disordered regions at the N- or C-termini often present difficulties with protein expression, purification, and crystallisation. Therefore, it is advised that disordered regions of a protein of interest are predicted before any structural studies are pursued [13]. Based on the data retrieved from computational predictions in this study, it appears that SACMV MP is intrinsically disordered, predominantly at the C-terminus. The normalised root-mean-square deviation NRMSD, which is indicative of the goodness of fit of the calculated data to experimental data (Dichroweb) (Figure S3) (<0.1 acceptable as a good fit), was calculated to be 1.041 for SACMV MP. This high NRMSD value further supports the prediction that MP contains long regions which are intrinsically disordered. Viral proteins often do not contain a well-defined secondary structure such as α-helix or β-strands but instead appear as random coils [23], further suggesting that MP is an intrinsically disordered protein (IDP) or is a protein with high disordered regions (IDPRs). Viral proteins such as the Nopp140 protein which is a nucleolar protein and shuttles between the nucleolus and cytoplasm in mammalian cells, and the UL56 protein from herpes simplex virus type 2 which is a membrane protein involved in vesicular trafficking, are illustrations of IDPs as most of their protein structure has been deemed disordered [19,24]. Proteins that are IDPs, or contain IDPRs, have the ability to take part in protein interactions with other proteins. These protein–protein interactions are especially facilitated in regions high in disorder [25]. Abutilon mosaic virus (AbMV) movement protein has been found to interact with HSP70 in *Arabidopsis thaliana* [26]. The interaction and binding of chaperone proteins to geminivirus movement proteins, in particular, suggest that these chaperones are needed for interaction with microtubules or cytoskeleton proteins.

Since structural studies on geminiviral MPs are absent from the literature, it is not known if ATP or other biomolecules bind to these proteins to form a complex in order to facilitate movement in the cell. In some geminiviruses, such as the Squash leaf curl virus (SqLCV), the MP traps the NSP–ssDNA complexes in the cytoplasm to move them to adjacent cells [26], suggesting the MP is capable of binding to other proteins. Since MPs are involved in the cell-to-cell movement of ss/ds DNA complexes, as proposed by the ‘couple-skating’ and ‘relay-race’ models, it can be predicted that MPs would bind ATP. The intracellular or intercellular movement of complexes along the plasma membranes would require energy. Indeed, the phosphate assay demonstrated that SACMV denatured MP was able to hydrolyse ATP (Figure 7), suggesting it must bind. However, whether this occurs with the native form of the MP in cells in planta is not known.

In order to confirm ATP binding, the presence of hydrophobic binding regions was investigated using an intrinsic mant-ATP assay. It can be proposed that in the denatured form in vitro, the hydrophobic regions of the MP are buried by hydrophilic sites, and therefore, mant-ATP molecules are unable to bind. Failure of the mant-ATP to bind to the denatured MP was observed by the decrease in quantum yield and lack of blue shift (λ_max_) (Figure 5). This suggests that in vivo, the MP may occur in a different conformation in its native form, allowing ATP binding to the predicted ATP binding site. ATP-binding domains on geminiviral MPs have not been predicted to date. Proteins that bind ATP are usually involved in cellular movement and membrane transport and are responsible for transporting substrates across extra and intracellular membranes [15]. Based on the ‘couple-skating model’ and the proposed functions of the domains of MPs, as well as in vitro ATPase activity demonstrated in this study, it is suggested that SACMV MP is able to bind ATP in order to localise to the cell periphery, but further in vivo studies need to be performed. A number of plant virus movement proteins have been shown to possess ATPase activity such as the MP of potato virus X and tobacco mosaic virus (TMV) [27]. More recent studies carried out on the geminivirus beta-satellite-encoded βC1 protein have also displayed ATPase activity [28]. Heat shock proteins such as heat shock protein 70 (HSP70) have also been shown to possess ATPase activity, and since HSP70 chaperones form interactive complexes with geminivirus MPs [8,9], they may perform the ATPase function during movement. It is proposed that the ATPase activity of SACMV MP and putative interactions of SACMV DNA–MP–NSP complex with HSP70 would facilitate cytoplasmic movement from the nucleus to the cell periphery. In planta, direct binding of ligands such as ATP to the native form of SACMV MP in the cell may not lead to conformational change. After the binding of a different ligand or DNA–protein complex to the MP in the cell, such as an HSP, the MP may change conformation, allowing the transfer of the DNA/DNA–protein complex to adjacent cells. This is supported by the active role of MP in cellular movement [8,9]. Size exclusion–high-performance liquid chromatography (SE–HPLC) was carried out on full-length MPs (data not shown); however, the correct retention time expected for protein elution was not attained. The protein appears to bind to the stationary phase of the column and elutes extremely late off the column, giving the incorrect retention time and resulting in incorrect molecular weight. It can be proposed that, due to the SACMV MP being highly intrinsically disordered and containing large hydrophobic regions, the protein remains bound to the non-polar stationary phase of the column, rather than moving through with the polar mobile phase, resulting in the protein eluting at the incorrect retention time [12]. The non-elution of the protein off the non-polar stationary phase of the SE–HPLC column further suggests that the protein may be aggregating and may form a high-order oligomer post refolding.

## 5. Conclusions

In conclusion, this research on the SACMV MP revealed, for the first time, the proposed secondary structure of a denatured refolded full-length monomeric geminivirus cell-to-cell movement protein in vitro. Due to the lack of attainment of soluble protein, denaturation-refolding was required. Results establish that the BC1-encoded MP from DNA-B is highly insoluble in solution and has a secondary structure which predominantly consists of β-strands. Its predicted large unfolded domain at the C-terminus presents some interesting speculations as to the function of the MP C-terminus. A study on Abutilon mosaic virus has revealed a homo-oligomerisation of the C-terminus of the MP and suggests that the virus possesses a host-plant species-independent capacity to bind to plasma membranes, likely occurring at the C-terminus. AbMV MP-mediated intracellular sorting is dependent on an ‘anchor domain’ (amino acids 117 to 180), which is necessary to traffic the MP complex to the cell periphery. We propose that SACMV MP has the ability to bind ATP in its native form due to ATPase activity, as demonstrated by the in vitro results herein. Viral MPs change in conformation in the presence of ATP and adopt a different conformation when denatured, compared with their native state. This suggests that, in the plant cell in vivo, native forms of the SACMV MP, or alternatively MPs that form a high-order oligomer, may undergo conformational changes when bound to biomolecules such as ATP. Indirect binding of other ligands with ATPase activity, such as HSP70, has been shown in other geminivirus studies, and recently *Arabidopsis* homologs HSP90 (AT5G56030) and mitochondrial HSP60 (AT3G23990) were demonstrated to interact with SACMV MP via Y2H and co-IP in cassava infected with SACMV. Since the MP has the proposed ability to bind ATP, further kinetic research may be carried out in future studies. In addition, further structural and molecular characterisation studies of geminivirus cell-to-cell movement proteins will unravel more in planta features of their functions.

## Data Availability

Not applicable.

## Supplementary Materials

The following supporting information can be downloaded at: https://www.mdpi.com/article/10.3390/cimb44060186/s1, Figure S1: (A) Annotated amino acid sequence of SACMV movement protein BC1 (Source: PSIPRED) and (B) the predicted secondary structure of SACMV movement protein BC1 (Source: Predict Protein); Figure S2: Vector map of expression vector pET28a (+) (5369 bp) containing kanamycin resistance and a multiple cloning site as well as multiple restriction sites (Source: Novagen online, 2017); Figure S3: Summary of the secondary structure content using the CONTILL alogothrim implemented in the Dichroweb server; Figure S4: The difference in nucleotide change for codon harmonization of SACMV MP for expression in *E.coli*.

## Abbreviations

MP	Movement protein
NSP	Nuclear shuttle protein
ANS	1-Anilino-8-naphthalene sulphonate-
mant-ATP	2′ (3′)- N- methylanthraniloyl- ATP
