# Supplementary figures and images for: Expression, Purification, and Characterisation of South African Cassava Mosaic Virus Cell-to-Cell Movement Protein
Source: Curr Issues Mol Biol. 2022 Jun 15;44(6):2717–29. doi: 10.3390/cimb44060186 (PMC9221656; doi:10.3390/cimb44060186)

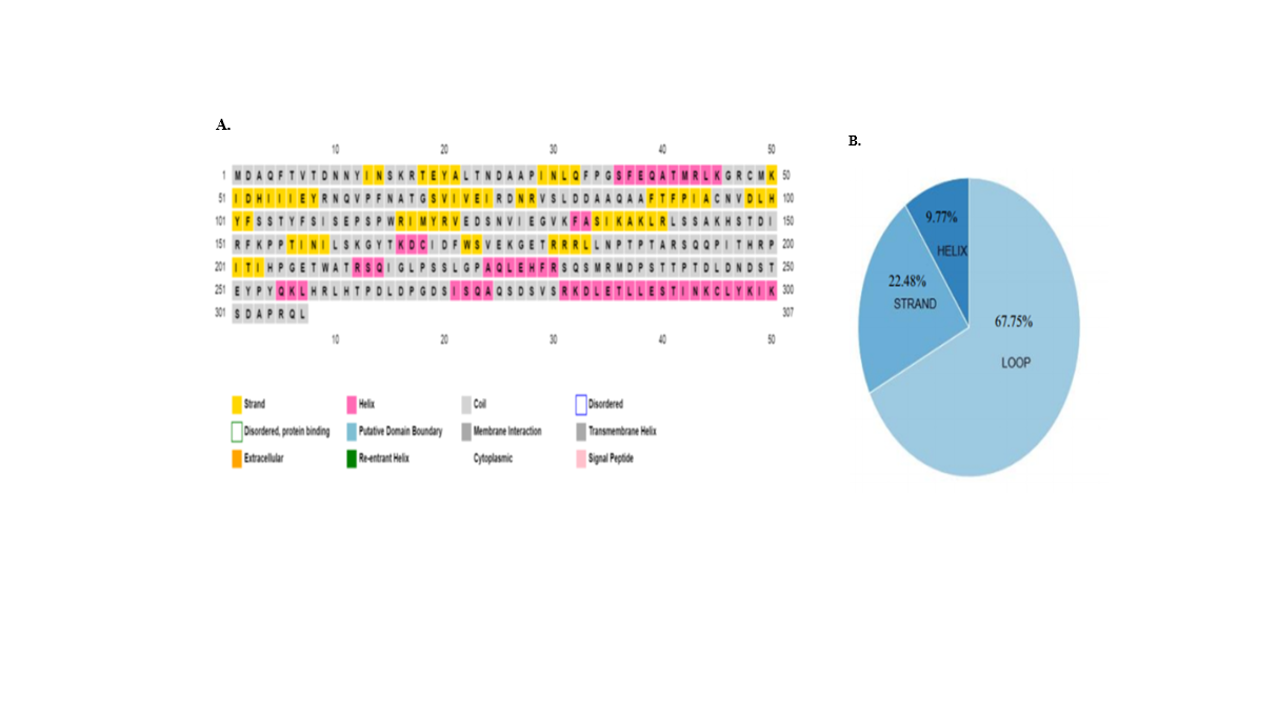

Supplement: Supplementary file 1 [file cimb-44-00186-s001.zip › Supplementary Data/Fig.S1.png]

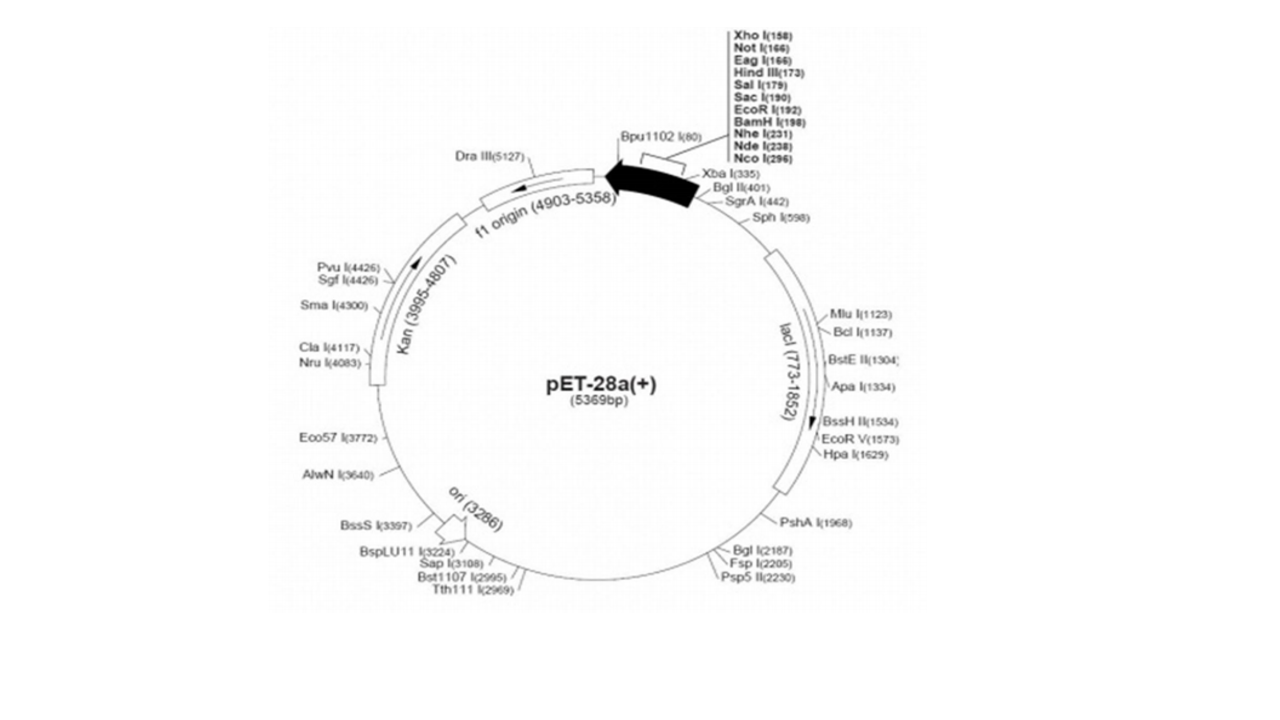

Supplement: Supplementary file 1 [file cimb-44-00186-s001.zip › Supplementary Data/Fig.S2.png]

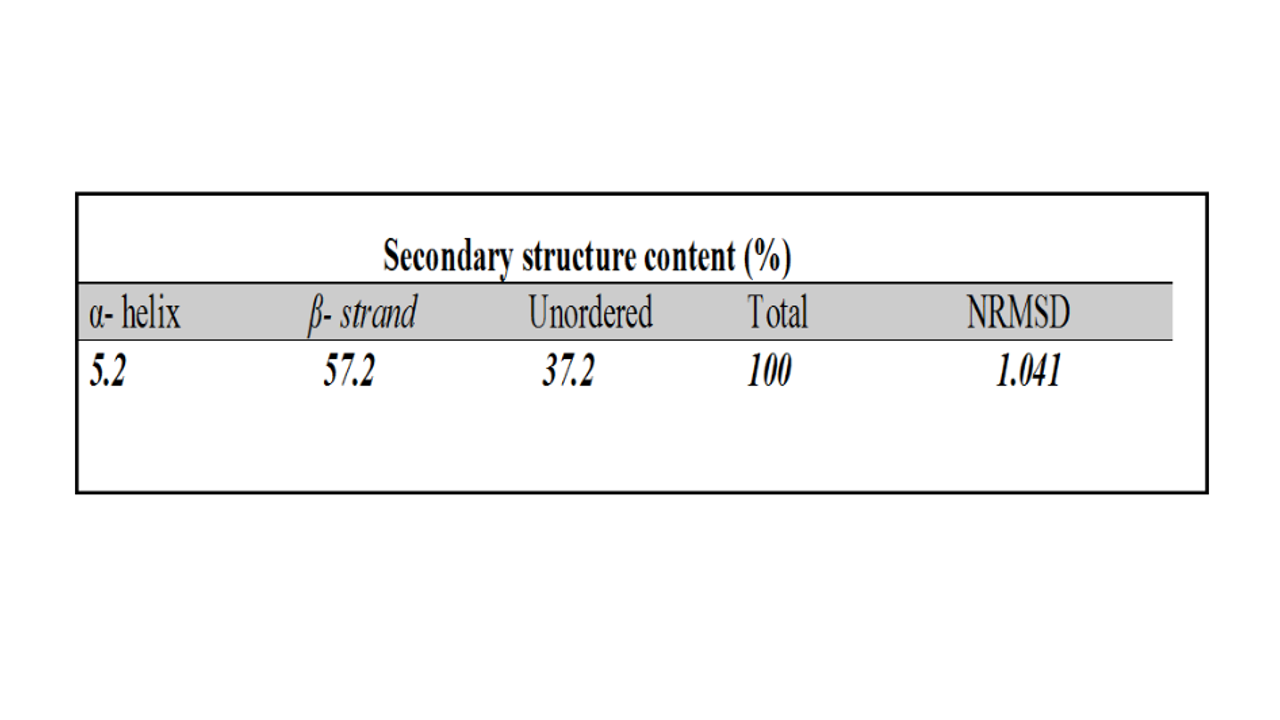

Supplement: Supplementary file 1 [file cimb-44-00186-s001.zip › Supplementary Data/Fig.S3.png]

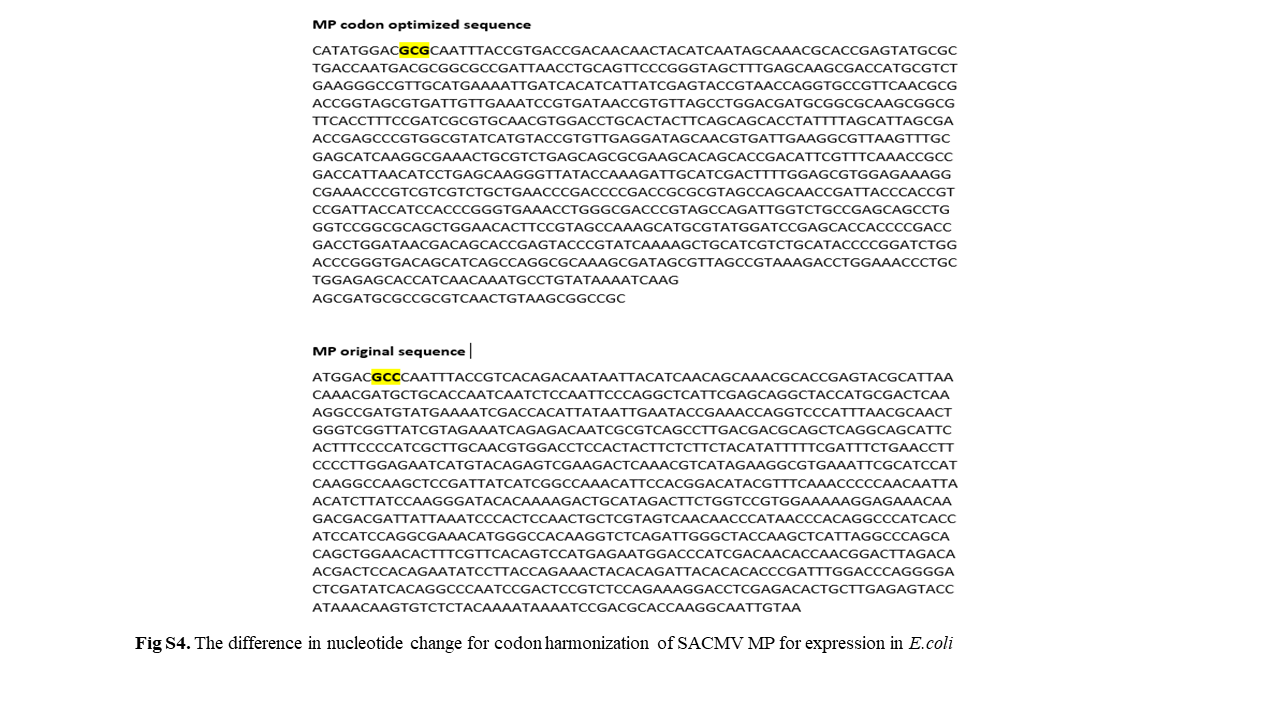

Supplement: Supplementary file 1 [file cimb-44-00186-s001.zip › Supplementary Data/Fig.S4..png]
